# Contribution of the Dolichos Lablab value chain to farmer's household livelihood assets in Tanzania

**DOI:** 10.1016/j.heliyon.2022.e11646

**Published:** 2022-11-17

**Authors:** Josephine J. Minde, Athanasia O. Matemu, Pavithravani B. Venkataramana

**Affiliations:** a Department of Food and Nutritional Sciences, Nelson Mandela African Institution of Science and Technology (NM-AIST), P.O. Box 447, Arusha, Tanzania; b Department of Sustainable Agriculture, Biodiversity and Ecosystems Management, Nelson Mandela African Institution of Science and Technology, P.O. Box 447, Arusha, Tanzania; c Department of Community Economic Development, The Open University of Tanzania, P.O. Box 517, Moshi, Tanzania

**Keywords:** Lablab value chain, Smallholder farmers, Livelihood assets, Underutilized legume

## Abstract

Lablab is a legume with multiple uses as food, feed, and organic fertilizer. However, it is underutilized, and its empirical contribution to farmers' livelihoods is limited. This study examined the Lablab value chain (LVC) relative to smallholder farmers' livelihood assets in Tanzania. Data on Lablab farming, its value chain, and its contribution to the farmers’ livelihood assets were collected from four districts of Tanzania known for Lablab production. The results showed that the LVC mapping comprised systems, actors, and interdependent activities, the key systems being input supply, tillage, intercropping, and marketing. Seed supply was through the recycling of the last harvest (48.8%), while intercropping (56.5%) and hand-hoeing (51.6%) were the primary cultivation systems, with farm gates and/or local markets serving as the marketing locations. The grown seeds were mainly black (62.3%) due to external marketability but were rarely eaten. Although farmers were the major chain actors, traders were highly active in the marketing segment, leaving farmers out of the bargain art protocol. Generally, Lablab production generated almost 90% of the farmers' revenue by multiple linear regression. Based on farmers' five asset categories, natural and human assets contributed 70% and 50% respectively, while there was less contribution from social, financial, and physical assets. The study recommends networking exposure to reliable marketplaces with good prices, improved seed availability, and financial services for Lablab farmers in Tanzania. To sustain the smallholder farmers' five livelihood assets from Lablab, researchers and policymakers must pay attention to the three linkages of the LVC.

## 1 Introduction

The agricultural sector provides adequate food and a livable wage for millions of people worldwide. According to the Food and Agriculture Organization (FAO, 2020), approximately 90% of the world's farms are owned and operated by smallholder farmers with more than 85% of these farms primarily supporting global livelihoods (Seville et al., 2011). However, household sustainability of rural livelihoods remains a major challenge (Adam and Osbahr 2019) and most smallholder farmers experience exacerbated food and livelihood challenges (Barnes et al., 2021; Kasolo et al., 2019; Baldermann et al., 2016). Consequently, the full potential of smallholder farmers is unrealized in different parts of the world. According to Nájera (2017), smallholder farm products are regularly and highly valued in the global value chains in developed countries. However, the farm product value chains in low-income countries are scarce and a majority of farmers have not shifted to modern production systems. Nord et al. (2021) and Larsson (2018), assert that smallholder farmers' practices are frequently overlooked in developing countries where there are few formalized links in various stages of agricultural value chains, particularly for underutilized and neglected crop species (Mabhaudhi et al., 2017).

Farming by smallholder farmers is of public interest as it supports the livelihoods of approximately 2.2 billion people worldwide (FAO, 2019; Nájera, 2017). However, in developing countries, data on smallholder farmers' interactions along the entire product value chains are often very scarce (Nájera, 2017). Therefore, a comprehensive value chain mapping exercise of farmers' crop production is necessary to provide more insight into the marketing and consumption of underutilized crops (Sperling et al., 2021; Larsson, 2018; Nájera, 2017; Eskola, 2005). According to Mabhaudhi et al. (2017) and Seville et al. (2011), quantifying the stages along crops’ value chains is vital for better chances and to overcoming efficiency threats.

Lablab (*Lablab purpureus* (L.) Sweet) is a drought-resistant and perennial legume with multiple uses and potential. However, there is limited data on its value chain relative to smallholder farmers' livelihoods, which may be overlooked or documented insufficiently (Nord et al., 2020, 2021; Minde et al., 2020; Maass et al., 2010). Despite being a traditional legume for many smallholder farmers in Tanzania, Lablab remains an underutilized crop in the country (Nord et al., 2020; Forsythe, 2019). The crop was adopted in Tanzania's northern areas in the 1990s due to threats of food and nutrition insecurity (AFASA, 2009). Similarly, unreliable rainfall and drought caused the failure of common crops and facilitated the adoption of Lablab. Ngailo et al. (2003), however, advances that the crop's dramatic price fall decreased its production. According to Forsythe (2019), the crop was re-introduced in northern Tanzania to support the existing system of one cropping which was continuously facing climate challenges, and to support the goals of improving smallholder agriculture for sustainable food production and poverty reduction (Nord et al., 2020; Forsythe, 2019). Besides, Bwalya (2005) previously revealed its profitability and good performance when intercropped with major staple crops such as maize in the Arumeru district, Arusha in northern Tanzania.

This reintroduction of Lablab in Tanzania is still limited by the lack of empirical data on its value chain relative to smallholder farmers' household livelihood assets, which is critical for its sustainability and farmer livelihood improvement. The value chain of smallholder farmers' produce requires attention because their farming has a significant impact on farmers' long-term social, food security, environmental, and financial benefits (Sperling et al., 2021; Muoni et al., 2019; Nicklin et al., 2006). Most studies, including those from Tanzania, have generally assessed Lablab's agronomical performance, but not its contribution to smallholder farmers' livelihood impacts (Minde et al., 2020; Morrison, 2019; Grotelüschen, 2014; Maass et al., 2010; Shetto and Owenya, 2007). Notably, determining the contribution of the Lablab value chain (LVC) to smallholder farmers' livelihood assets can ensure the crop's sustainability and wider adoption. Therefore, this study assessed the LVC among Tanzania's smallholder Lablab farmers in the northern zone districts of Babati, Arumeru, Same, and the central zone of Mvomero. The study further evaluated the LVC in terms of its contribution to the farmers' household livelihood assets like natural, social, human, and financial. The livelihood index was computed based on the assets produced as a result of participation in Lablab production to further explain its effects considering different socioeconomic variables. The findings are critical in ensuring long-term Lablab farming for smallholder farmers' livelihood development as well as providing a link in global crop value chains. Besides, understanding the LVC among smallholder farmers relative to their livelihood can provide insight into future intervention opportunities and challenges.

## 2 Materials and methods

**Figure 1:**
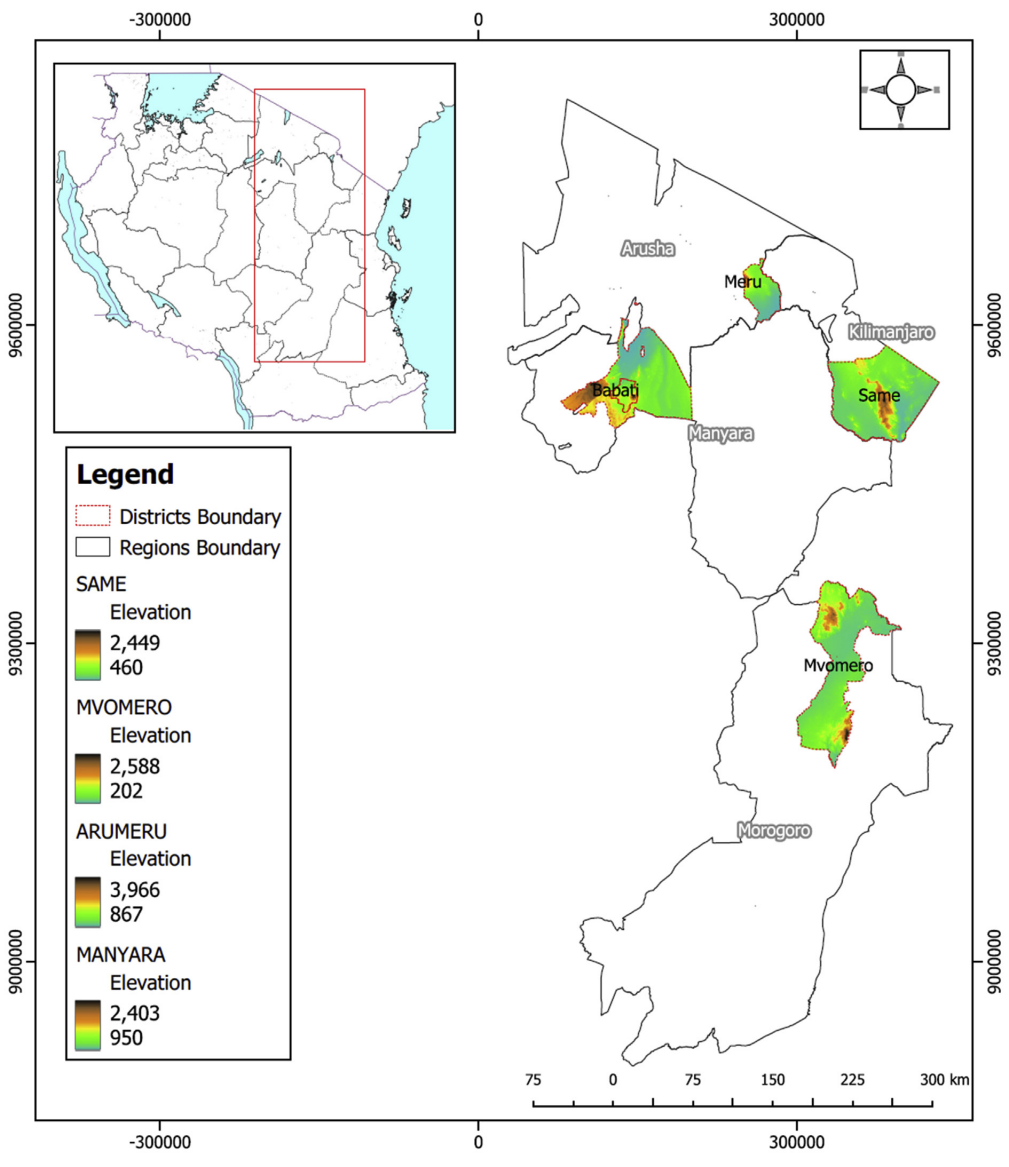
A map showing four agro-ecological zones growing Lablab in Same, Mvomero, Arumeru, and Babati districts in Tanzania.

The study was conducted in the agroecological zones of Arumeru, Babati, Same, and Mvomero districts in Arusha, Manyara, Kilimanjaro, and Morogoro regions respectively in Tanzania (Figure 1). The elevations in the four districts ranged from 867 to 3966, 950 to 2403, 460 to 2449, and 202–2588 m above sea level, respectively. The selected areas all support Lablab production despite the variations caused by climatic factors such as temperature, humidity, and precipitation systems (Shetto and Owenya, 2007).

The researcher employed a sample space of 386 study participants consisting of 344 farmers (86 from each district), and 40 buyers/traders (10 from each district). The researcher selected the farmers at random from a list of Lablab growers provided by village extension officers who doubled up as the key informants in the research. The buyers and farmers were selected based on their involvement in the Lablab marketing and cultivation respectively. The research consisted of field observations and in-depth face-to-face interviews using a structured questionnaire to collect data on; i) the socio-demographic characteristics of the study participants, ii) the LVC statements from production to consumer, and iii) the contribution of LVC to farmers’ livelihood assets.

### 2.1 Data analysis

All analyses were carried out using the R-Statistical software (Version 4.11). Data on names of districts and sex of participants were assigned values such as yes (1) or no (0) and inferentially analyzed before being incorporated into the multiple regression model. Value chain analysis was conceptualized based on the chain map proposed by Tadesse and Bakala (2018). The framework was developed with three components namely systems, actors, and activities, each described through their different phases up to the final consumers. Descriptive data such as percentages were used to analyze the participation of traders in the LVC while the farmers' household livelihood assets were measured using indicators developed from the chain framework. According to de Satgé et al. (2002), the formulation of indicators assists in measuring, counting, describing, and presenting an individual's or a community's reflection on a given topic. The indicators in this study presented the expected relationships that Lablab farmers had and what they could gain for their livelihoods (Appendix I). Tailored LVC indicators were used to measure the farmers' household livelihood assets. On each LVC indicator, each farmer had a chance to score 1 (yes) or 0 (no); yes, if he/she had a chance to possess an asset resulting from Lablab production, and no if otherwise. The total score on each indicator (asset), average score per indicator, total average scores per asset category, and average score per asset category were then calculated to determine the contribution of the LVC to farmers' livelihood assets.

The average score per indicator was obtained by dividing each asset's total score indicator by the total number of farmers interviewed (344). The average score for each category of assets was obtained by summing up the total scores of all the indicators and dividing the sum by the number of indicators in the category. Furthermore, the average for each of the five assets was summed up, and the result was divided by five (total number of the asset categories) to obtain the average asset score of farmers in the study area.

Factors influencing the farmers’ livelihood assets were determined using multiple linear regression. The livelihood index (dependent variable) was created by adding all assets resulting from Lablab production per individual to determine the relationship with selected socio-economic factors including sex (male or female), age (in years), marital status (married or not), farm size (hectares), seed sources (bought or not), farming style (sole or intercropped), harvesting style (uprooting whole or pick dry pods), seasonal harvest (yield), identified sales destinations (yes or no), farm gate sales (yes or no), income from Lablab sales, and income from other sources. The selected variables were checked for normal distribution using the correlation coefficient (r-value) to avoid multicollinearity before analysis and to minimize the occurrence of high intercorrelations (closeness of association) among two or more independent variables in the multiple regression model (Paudel Khatiwada et al., 2017a, Paudel Khatiwada et al., 2017b).

## 3 Results and discussions

### 3.1 Lablab value chain characteristics

**Figure 2:**
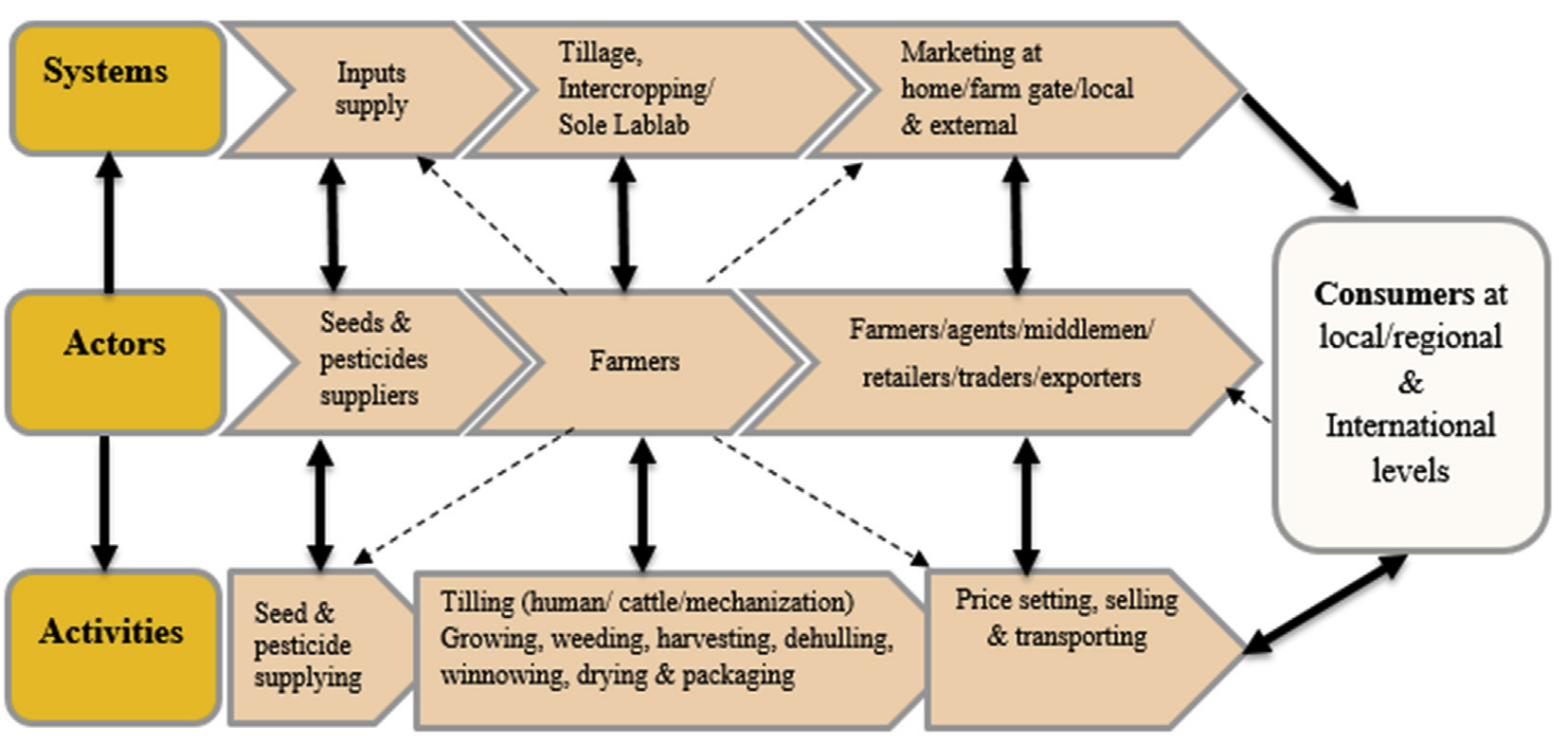
Value chain mapping for Lablab crop in Tanzania (Tadesse and Bakala, 2018). Key: [] Strong existing channels [] Weak existing channels [] Normal existing channels.

Understanding the nature of agricultural production chains is critical for knowledge generation for marketing channels (Muoni et al., 2019; Tadesse and Bakala 2018). Figure 2 illustrates the mapped LVC identified in Tanzania along with their interlinkages.

The LVC in Tanzania is largely composed of three categories; systems, actors, and activities (Figure 2). The LVC is highly informal, lacking integrated formal linkages, a characteristic of an underdeveloped value chain in most low-income countries (Nájera, 2017). Detailed information on the LVC categories is provided in the sub-sections 3.1.1 to 3.1.3.

#### 3.1.1 The Lablab value chain systems

**Figure 3:**
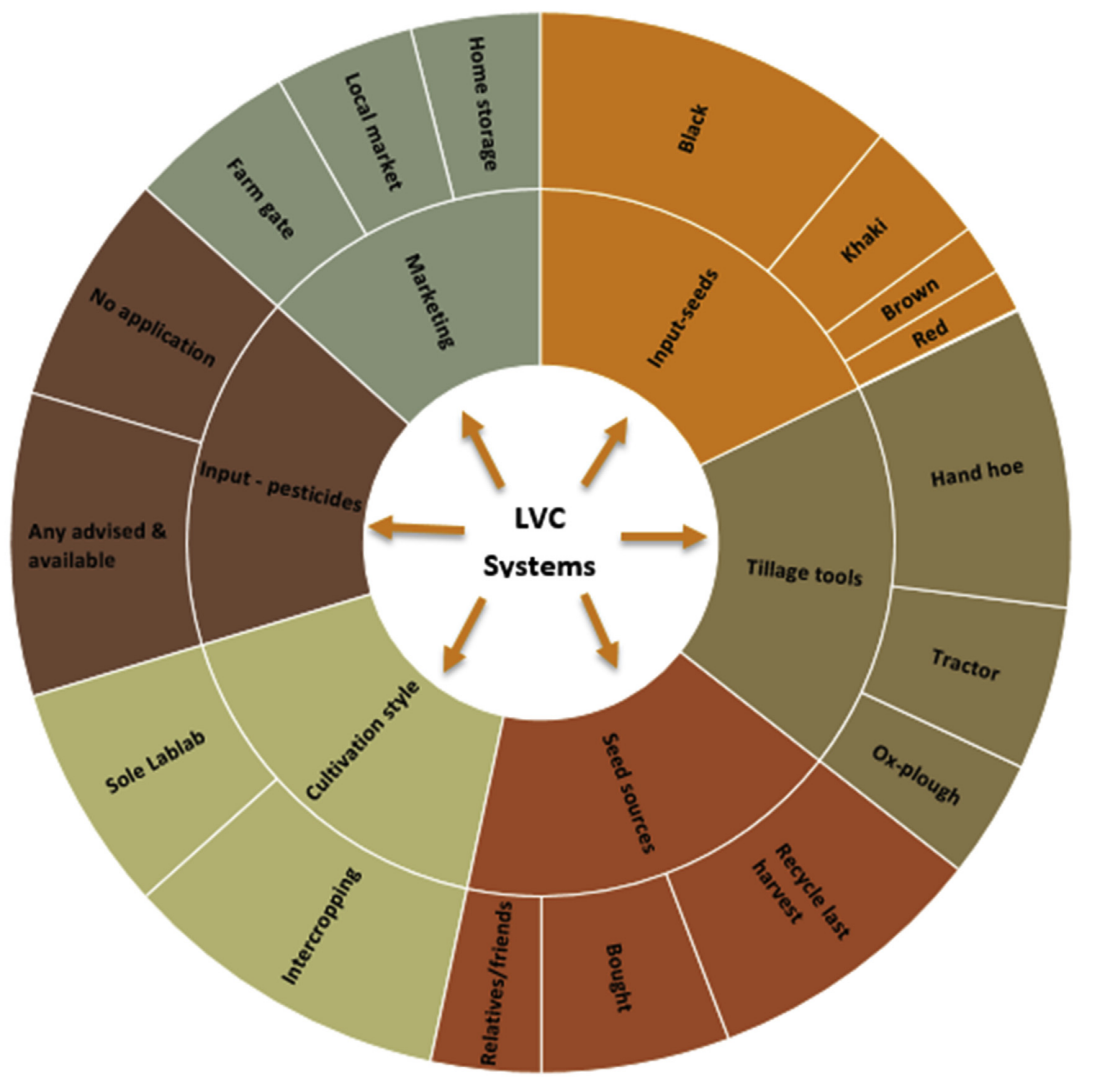
The Lablab value chain systems and their subdivision magnitude in Lablab crop growing districts in Tanzania.

The Lablab production system in the study areas includes input supply, tillage, sole cropping or intercropping, and marketing (Figure 3). Input supply encompasses seed and pesticide suppliers. For the study participants, the primary seed supply sources were relatives/friends, buying from local markets, and recycling from previous harvests (Table 1). Most of the farmers also recycled seeds from previous harvests (48.8%). The black accession was the most cultivated seed in Arumeru (93.3%), Same (55.9%), Babati (53.8%), and Mvomero (45.9%) districts (Table 1). On the other hand, the khaki, brown, and red accessions were less grown, unfamiliar across the study locations, and had a low market value (Figure 3). The black accessions were mostly cultivated (62.3%) due to their marketability outside Tanzania. According to Grotelüschen (2014), the black accession Lablab is well-marketed, and farmers are usually motivated by what is more significant in terms of profit margins (Morrison, 2019).

**Table 1:** Lablab value chain (n = 344).

Variable	Districts				Average %
	(i) Arumeru %	Babati %	Same %	Mvomero %	
**Accessions color**					
Black	93.5	53.8	55.9	45.9	62.3
Red	1.1	11.0	17.0	0.0	7.3
Brown	0.0	2.1	9.4	22.8	8.6
Khaki/Cream yellowish	5.4	33.2	16.5	31.4	21.6
White	0.0	0.0	1.1	0.0	0.3

**Source of seeds**					
Relatives/friends	24.4	5.8	10.5	34.9	18.9
Buying	30.2	76.7	38.4	50.0	32.3
Recycling the last harvest	45.3	17.4	51.2	15.1	48.8

**Pesticides used**					
Any as advised and available	89.3	67.3	68.2	12.9	60.3
No application	10.6	32.9	31.9	88.4	39.8

**Reasons for not using pesticides**					
Causes health problems	8.4	9.1	0.0	5.1	5.7
No specific pesticide for Lablab	36.1	31.1	24.2	40.4	33.0
Costly	55.4	59.7	75.8	54.5	61.4

**Sources of pesticides?**					
Agro-shops	82.7	89.3	74.8	84.4	82.8
Local markets	17.3	0.0	23.3	4.2	11.2
Cooperative society	0.0	0.0	0.0	4.2	1.1
Relative/friends	0.0	10.7	2.9	7.3	5.1

**Tillage tools used**					
Hand hoe	44.4	37.1	52.4	72.4	51.6
Ox-plough	26	47.4	7.8	0.0	20.3
Tractor	29.5	15.4	39.8	27.6	28.1

**Cultivation systems**					
Pure/Sole Lablab	32.5	26.8	51.2	45.9	35.0
Intercropping with other crops	67.4	73.3	36.5	48.8	56.5

**Harvesting procedures**					
Uprooting dried crop	20.8	29.1	40.7	10.4	25.3
Dried ponds picking	67.3	65.2	55.6	58.1	61.6
Green beans picking	11.9	5.3	3.7	31.5	13.1

**Marketing**					
Farm gate	48.8	19.8	23.0	4.3	29.0
Local market	31.6	44.8	33.6	5.0	24.0
Home Storage	14.7	32.6	28.7	13.4	22.2

Pesticides are also used as inputs in Lablab farming in Tanzania. About 60.3% of interviewed farmers got advice and purchased available pesticides in agri-shops (Table 1). However, the pesticides and insecticides used for Lablab production were the same as those used for other crops. This implies the inability to control specific insect pests that affect the Lablab crop. Besides, the pesticide shops/suppliers were primarily located in urban and semi-urban areas, presenting accessibility challenges for farmers in remote areas (Shetto and Owenya, 2007).

As a result, 61.4% of the interviewed farmers were worried about pesticide costs, and 39.8% were unable to use them during the 2020 farming season (Table 1). Insect and disease control is critical to ensuring profitable Lablab output because the crop is greatly affected by pests from planting to podding (Ewansiha et al., 2016). The present study suggests that agriculture officers should support smallholder Lablab farmers in this regard to increase agricultural production, income, and food security for improved rural livelihoods (Popoola et al., 2019; Cullis and Kunert, 2016).

The Lablab farmers primarily used the hand-hoe, ox-plow, and tractors as tilling tools (Figure 3). Hand-hoe farming was most popular in Mvomero (72.4%) and Same (52.4%) districts (Table 1). Similarly, most smallholder farmers in Kenya tend to use hand hoes while the medium-sized landholders and large-scale farmers tend to employ oxen and tractors respectively (Rusike et al., 2013). Farming was mechanized for 28.1% of the study participants on average (Table 1). Mechanized farming is one of the possible solutions to changing traditional agricultural practices to enable the participation of smallholder farmers in global value chains (Rashid et al., 2019; Nájera, 2017). Multiple regression results revealed that both cultivation systems (sole and intercropping) contributed significantly (p ≤ 0.01) to the farmers' livelihoods (Table 3). Lablab intercropping with major staple foods such as maize, cassava, and sunflower was most widely utilized in Babati (73.3%) and Arumeru (67.4%) districts (Table 1). Similarly, maize and Lablab beans are often intercropped in Kenya (Rusike et al., 2013) and other regions in Africa (Ewansiha et al., 2016). Since Lablab is a cover crop, intercropping it with other crops can reduce tillage and save on labor (Forsythe 2019; Shetto and Owenya, 2007). Other crops grown with Lablab in the study areas included sorghum, millet, and bananas which also serve as food and cash crops (Table 1).

Lablab marketing system is another component in the LVC that was accomplished at the farm gate, local markets, or at home in the present study (Figure 3). Farm gate selling was particularly common in the Arumeru district which entailed a sale of more than 40% of the produce (Table 1). The farmers preferred this outlet because it excluded transportation costs unlike selling in the local market which requires traveling to urban centers and markets once or twice every week. In the northern region, the traditional local markets for Lablab beans included Hedaru, Kwasakwasa, Himo, Kikatiti, Ngaramtoni, and Mbauda. Most of the Lablab purchased from farmers also got transported to Kenya. Such external markets have largely motivated the Labalab farmers in Tanzania to expand their production for profits (Morrison, 2019). According to Nicklin et al. (2006) research on marginal/underutilized legume markets can lead to food and nutrition security as well as revenue for farmers. In the present study, Lablab income significantly contributed about 90% of farmers’ income (p ≤ 0.001) (Table 3). Lablab is recognized as a great source of revenue for farmers in Same and Arumeru (Same District Council, 2018; Shetto and Owenya 2007). Similarly, Lablab has higher prices than common beans in the northern zone of Tanzania (Shetto and Owenya, 2007). Other crops also contributed significantly (p ≤ 0.001) to farm income and accounted for over 40% of the total farm income (Table 3). This is commendable because farmers with diverse sources of income have higher incomes for their living, as stated by Adam and Osbahr (2019).

#### 3.1.2 Lablab value chain actors

Farmers, input suppliers, and traders/middlemen are the main actors in the LVC in the study areas (Figure 2). However, smallholder farmers are the major actors in Lablab production, taking part in a variety of activities along the value chain. According to Rusike et al. (2013), farmers usually engage in a variety of farming operations that help them to better their living conditions. The effect of gender was significant (p ≤ 0.05) (Table 3), with the males clearing the land and women and children getting assistance from men to plant Lablab.

Most inputs suppliers were seldom found with Lablab seeds in their Agro-shops, implying that a few of the study participants (32.3%) bought Lablab seeds from the local markets. Although farmers in rural areas, usually buy seeds from village markets (Rusike et al., 2013), farmers in the present study were observed to rely on recycling Lablab seeds from previous harvests (Figure 3) or obtaining the seeds from research institutions such as the Selian Agricultural Research Institute (SARI) in Arusha.

A previous study by Shetto and Owenya (2007) also reported that farmers from Arusha, Manyara, and Kilimanjaro regions were beneficiaries of Lablab seeds from SARI. Inorganic fertilizers were not delivered by input suppliers because the crop has a higher symbiosis ability with nitrogen-fixing bacteria that enriches the soil (Popoola et al., 2019).

**Figure 4:**
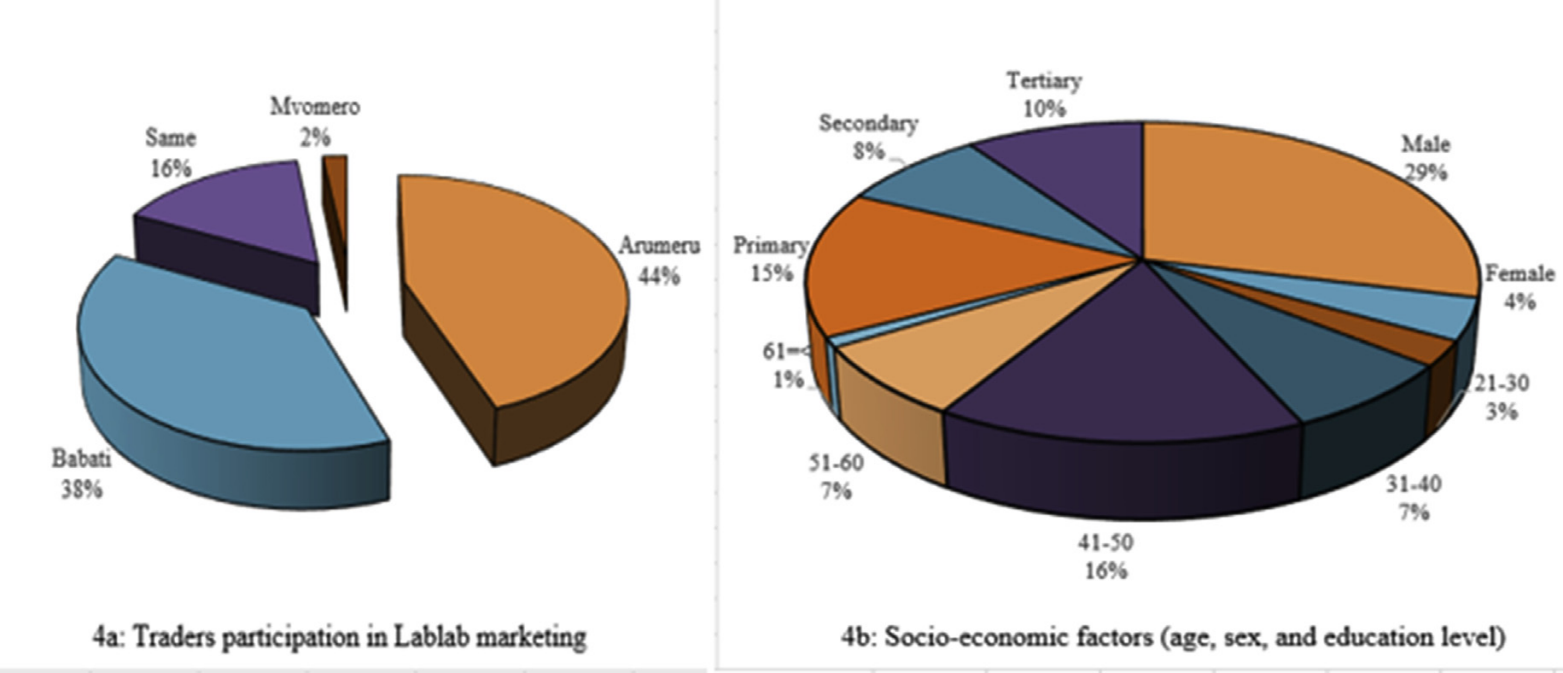
Value chain actors – traders (n = 40).

Traders were the key actors in the marketing system of the LVC (Figure 4). Noteworthy, the term ‘traders’ is often used interchangeably with buyers, agents, middlemen, and retailers. In Babati, Tanzania, the same scenario was recorded in a sunflower value chain (Larsson, 2018). In the LVC, traders were middlemen between farmers (producers) and retailers or consumers. Moreover, the traders acted as agents for internal (farm gates/local) and external Lablab markets as well as input providers to farmers (Figure 2). The findings revealed that traders' participation in Lablab marketing varied (Figure 4a), and was related to market reliability, particularly external market consistency (Nord et al., 2020; Forsythe, 2019). The participating traders were almost all young men (Figure 4b) capable of engaging in a variety of business activities (Darnhofer and Vienna, 2016).

Lablab was less cultivated in Mvomero, resulting in fewer traders (Figure 4a). Unreliable Lablab markets and low Lablab prices of less than half a US dollar (600–1000 TZS) per kilogram were among the identified reasons for its low production in Mvomero. As a result, most farmers in the district grew Lablab in their backyards or small areas close to their homes for food. The study generally demonstrated that traders' involvement in the Lablab farming business fueled farmers' interest in farming since traders were key connectors to external markets. The known external market for Lablab beans is Kenya (Forsythe, 2019). The present study confirms that farmers' markets in Tanzania are still dominated by traders who play an important part in the art of bargaining, rather than the farmers (Darnhofer and Vienna, 2016; Eskola, 2005). Even in places where farmer associations exist like in the Arumeru district, none were observed to have a concern about underutilized crops like Lablab. However, cooperative unions/societies seemed capable of establishing reliable marketing mechanisms in which farmers are the owners and can negotiate higher prices for their products. According to Shetto and Owenya (2007), cooperative unions/societies can sustain efficient marketing systems in which farmers are the owners and have the opportunity to negotiate prices for their products.

**Figure 5:**
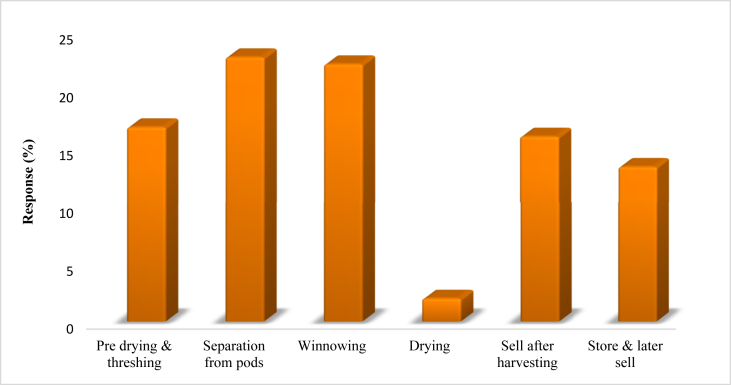
Harvesting procedures for Lablab beans in the selected Lablab growing districts in Tanzania.

**Figure 6:**
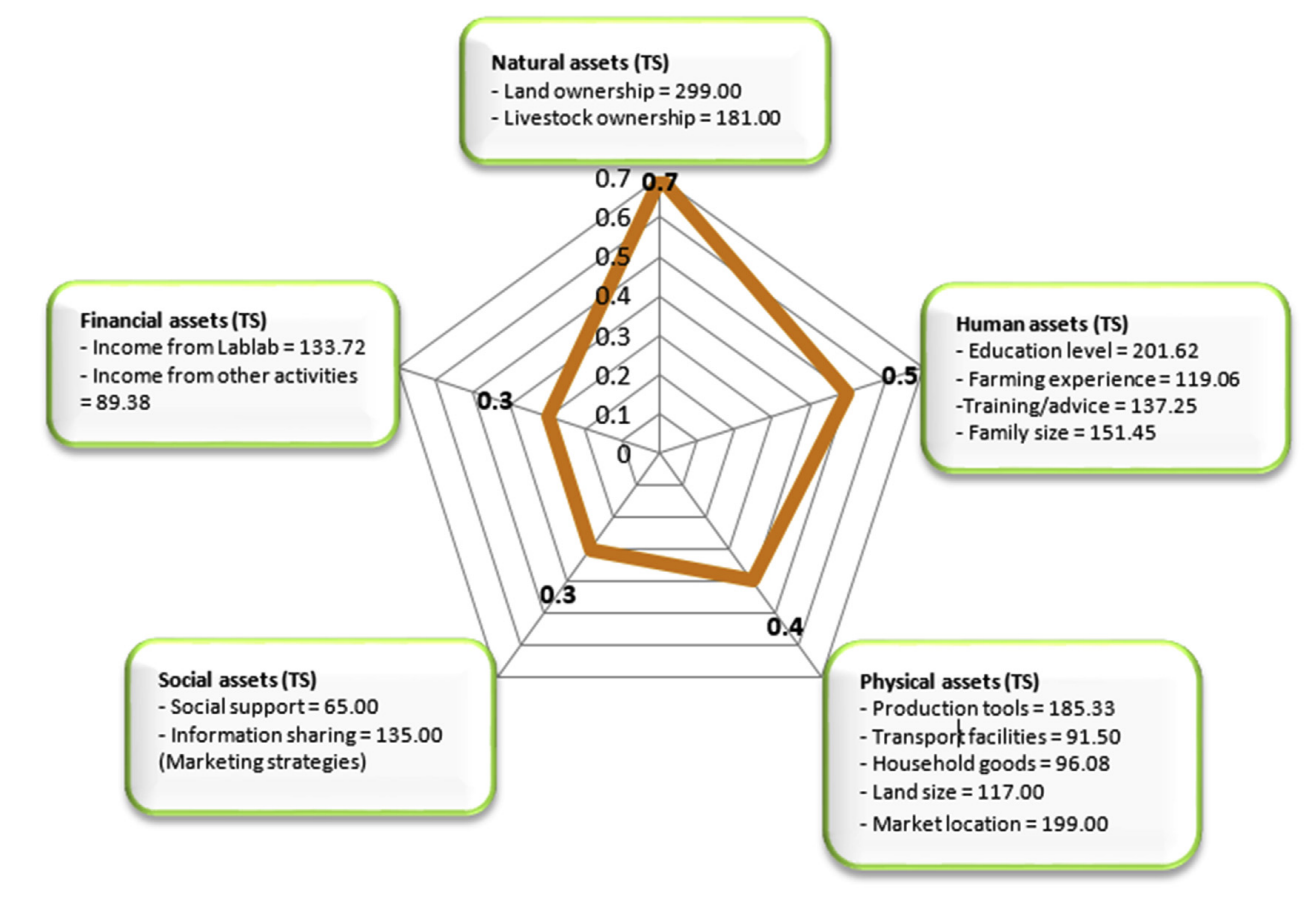
Livelihood assets spider pentagon represents the average assets score per asset category in the study. The assets show the total scores measurement of each developed indicator from farmers' households' characteristics.

The traders/middlemen/agents interviewed in the present study had more than five years of expertise in Lablab marketing. They were largely involved as negotiators in Lablab marketing, causing farmers to sell their produce at below-market prices. The traders' propensity of purchasing the beans at the farm gate immediately after harvesting (Figure 5) accounted for the cheap prices. According to the findings, the farmers sold their Lablab beans at cheap rates due to a lack of experience and social networking (Figure 6). The findings further revealed that the challenges experienced during storage such as insect infestations were among the variables that influenced the quick sale of produced beans due to lack of proper storage equipment, and insufficient storage experience and abilities. As a result, the traders were compelled to sell their products at lower prices at the farm gates immediately after harvesting.

#### 3.1.3 Lablab value chain activities

The activities along the LVC and the overall networking activities were diverse as conceptualized in Figure 2. Although Lablab farming included sole/intercropped planting (Figure 3), intercropping was mostly practiced by the smallholder farmers while their large-scale counterparts engaged in sole cropping. The farming season began in late May or early June at the end of the long rainy season with the Lablab seeds being sown 2–3 weeks after the maize seeds germinated and attained approximately 15 cm in length. Planting Lablab later reduced entanglement when intercropped with cereals. This is because Lablab is a climbing legume that can obstruct the physiological growth of cereal crops when planted at the same time (Nord et al., 2020; Mthembu et al., 2017; Dahmardeh et al., 2009; Eskandari et al., 2009).

Lablab weeding was recorded to require less labor because it is a cover crop that reduces weeding costs and results in significant labor savings for farmers (Silva and Delate, 2017). Apart from reducing weeding costs, Lablab plants can also reduce soil erosion and evaporation (Silva and Delate, 2017; Shetto and Owenya, 2007). Lablab biomass that covers the soil also increases the availability of fodder for livestock keepers (Mthembu et al., 2017). The results concerning the management of insect pest damage activities identified poor pest control as a critical challenge (Figure 2). On average, about 61.4% of the farmers found it expensive to use pesticides during Lablab farming while 33% did not know the specific pesticides for Lablab crops (Table 1), leading to poor yield and subsequently, dislike for Lablab farming.

Harvesting was done manually by picking the green beans and dry pods or uprooting the entire plant (Table 1). Green bean harvesting was more common in the Mvomero district (31.5%) (Table 1). Harvesting by picking dry pods was a common practice (61.6%) across the study sites, particularly in Arumeru (67.3%) and Babati (65.2%) districts (Table 1). Owing to genetic and environmental conditions, Lablab beans do not dry all at once, making harvesting by picking up dry pods twice/thrice tiresome and costly (Minde et al., 2020; Cullis and Kunert, 2017; Kimani et al., 2012). During harvesting, a worker was paid TZS 1500 (about US$ 0.64) every day or TZS 5000 (US$ 2.16) per sac, thus increasing the cost of harvesting. According to Grotelüschen (2014), Lablab harvesting is a very expensive, laborious, and time-consuming activity.

Post-harvest processing and storage practices were noted to be varied (Figure 5). Generally, the harvested Lablab were pre-dried, threshed manually by beating with a stick, winnowed, and dried. The large-scale farmers, on the other hand, used tractors to separate the beans from the pods, followed by hand-winnowing with ‘*ungo’* or blowing by wind, and finally drying them in the sun/air. Bean storage was inadequate and bruchid beetles caused substantial damage. Pengelly and Maass (2001) document that insects cause significant damage to Lablab beans, necessitating careful storage measures.

### 3.2 Lablab value chain contribution to household livelihood assets

Lablab production benefited the farmers considerably. Lablab was grown even in semi-arid areas as observed in the Same district (Same District Council, 2018). In Tanzania, Lablab can be dubbed a climate-smart crop because it is a drought-tolerant and perennial crop that contributes to agricultural conservation. Lablab was reintroduced in the country to provide additional revenue to smallholder farmers due to its capacity to survive adverse climatic conditions such as unreliable rainfall which affects popular crops such as maize and common beans (Forsythe, 2019; Grotelüschen, 2014; Shetto and Owenya, 2007). Literature demonstrates that farmers' participation in Lablab production benefits their families in a variety of ways (Minde et al., 2020; Grotelüschen, 2014; Shetto and Owenya, 2007). According to de Satgé et al. (2002), an individual can own five types of livelihood assets: natural, social, human, physical, and financial assets. The LVC indicators identified in the present study were related to the different categories of livelihood assets after the farmer engaged in Lablab production. The benefits of Lablab production provided in Table 2 as natural, human, financial, physical, and social assets demonstrate how much Lablab contributes to smallholder farmers' livelihoods.

**Table 2:** Developed indicators from LVC used to measure farmers’ household livelihood.

Livelihood asset categories	Indicator	Total scores (n = 344)	Average score per indicator	Total scores per asset category
**Natural assets**	(a) Land ownership (b) Livestock ownership	299.00	0.87	1.40
		(c) 181.00	0.53	
**Human assets**	(a) Education (b) Farming experience (c) Training/Advice (d) Family size	201.62	0.59	1.78
		(e) 119.06	0.35	
		(f) 137.25	0.40	
		(g) 151.45	0.44	
**Physical assets**	(a) Production Tools	185.33	0.54	2.01
	(b) Transport facilities	91.50	0.27	
	(c) House and its goods	96.08	0.28	
	(d) Nearest market	117.00	0.34	
	(e) Usage of Pesticides	199.00	0.58	
**Social Assets**	(a) Social support from Friends/Relatives	65.00	0.19	0.58
	(b) Communication to networks	135.00	0.39	
**Financial Assets**	(a) Lablab income	133.72	0.39	0.65
	(b) Other sources income	89.38	0.26

**Table 3:** Relationship between the Livelihood Index and social-economic factors.

Variables	Coefficient	Standard Error	P-value	Conf. Interval 95% Low Upper
Sex	0.145**∗**	0.063	0.023	0.020–0.269
Age	0.012**∗∗∗**	0.003	0.000	0.006–0.018
Marital status	0.057	0.039	0.147	-0.020–0.134
Farm size	0.042**∗**	0.019	0.029	0.004–0.080
Seed sources	0.081**∗**	0.039	0.038	0.005–0.157
Cultivation systems	0.130**∗∗**	0.038	0.001	0.055–0.206
Harvesting style	0.059	0.070	0.400	-0.079–0.197
Harvest per season	0.065**∗∗**	0.021	0.002	0.023–0.107
Sell destinations	0.320**∗∗∗**	0.036	0.000	0.249–0.392
Farm gate	0.106**∗∗**	0.035	0.003	0.366–0.176
Income Lablab	0.909∗∗∗	0.087	<0.001	0.737–1.081
Income others crops	0.399∗∗∗	0.105	0.000	0.192–0.606
Number of obs	344			
F (11, 333)	360.25			
Prob > F	0.0000			
R-squared	0.9225			
Adj R-squared	0.9199			
Root MSE	0.61273			

Statistically significant level: ∗∗∗p ≤ 0.001; ∗∗p ≤ 0.01; ∗p ≤ 0.05.

In general, measuring rural livelihoods based on income criteria is not worthwhile; nevertheless, applying the five capital assets in understanding rural lifestyles relates to livelihood security and is advantageous (Halberg and Muller 2012). The following paragraphs demonstrated the influence of LVC on each asset and its contribution to the farmers' livelihoods.

Land and cattle ownership is tangible natural resources that can bring changes in people's lives (de Satgé et al., 2002). Lablab farmers in the present study owned land and livestock (Appendix 1) as natural assets that contributed to higher income, work opportunities, food/fodder, and soil conservation. Land generated 87% of the average indicator score, while livestock contributed 53% (Table 2). This asset delivered a total score per asset of 70% to farmers' lives (Figure 6). Moreover, a majority of farmers (56.5%) could conduct intercropping of Lablab beans with maize or sunflower (Table 1) and acquire food and fodder for their livestock on the same piece of land. Previous results show that intercropping Lablab with maize usually results in high grain yields and high-quality feed for cattle (Mthembu et al., 2017). Besides, intercropping legumes and grains boost farmers' land productivity (Halberg and Muller, 2012).

The present results revealed that the Lablab intercropping technique controlled weeds, which saved labor and minimized soil erosion because Lablab is a cover crop. It also enhanced farmers' lands as an organic fertilizer. As per Halberg and Muller (2012), bio-fertilized land produces crops with a high market value which increases income in rural communities. Lablab leaves, on the other hand, were used as livestock feed. In comparison to other legumes, Lablab leaves contain a higher protein content (up to 28%) which feeds calves and increases milk supply (Minde et al., 2020; Aganga and Tshwenyane, 2003). Despite their small land area, the identified potentials of this crop benefited the farmers directly or indirectly. The findings showed significant effects of the smallholder farms (p ≤ 0.05) and cultivation systems (p ≤ 0.01) on the farmers' lives (Table 3). Obtaining such data on smallholder farming has broader implications for their agricultural activities in connection to their lives (Popoola et al., 2019; Wijk et al., 2018; Darnhofer and Vienna 2016; Owenya et al., 2011).

Human assets are intangible assets such as the ability to work, household labor-power, knowledge, and skills gained through generations or via observations to better living conditions and seek out new chances (FAO & ILO, 2009). Collectively, education, agricultural experience, training/advice, and family size assisted the Lablab farmers' households to increase output and income for livelihoods. According to the findings, education had a positive impact on the farmers' lives, contributing around 59% on average per indicator (Table 2). The age and sex of the Lablab farmers were used as the baseline variables in this study, whereby sex had a statistically significant impact (p ≤ 0.05) on the Lablab business. This means that both male and female farmers participated in Lablab production (Table 3), an indication that gender relations were important in Lablab crop production. Gender participation in direct and indirect Lablab conservation agriculture was previously estimated to be roughly 900 and 300 families respectively in the districts of Arumeru, Karatu, and Bukoba (Bwalya, 2005).

The present study demonstrated that the number of years spent cultivating Lablab had a significant impact on livelihoods as farmers' ages had a statistically significant impact (p ≤ 0.001) on their livelihoods (Table 3). This is probably because the older farmers were more skilled and experienced in Lablab farming, resulting in more prosperity for their families. The study also showed that the training/advice indicator results contributed about 40% of the average score to farmers' livelihoods (Table 2). The farmers were observed to seek advice from their elder parents/relatives or other farmers with greater expertise. In most cases, the smallholder farmers in rural areas received information such as marketing opportunities through their fellow farmers (Tefera, 2006).

Also assessed as an indicator under human assets was the family size which contributed about 44% to farmers' lives (Table 3). This suggests that farmers' households had a sufficient number of family members who actively participated in Lablab farming. Under normal circumstances, family members of working age can provide substantial labor for agricultural activities (FAO & ILO, 2009). Generally, all indicators assessed under human assets contributed approximately 50% to farmers' livelihoods (Figure 6).

The farmers' families benefited from financial assets, or capital/revenue generated through Lablab production, which contributed about 90% of their income (Table 3). Since the black Lablab beans are highly marketable outside Tanzania, farmers in the study areas were highly attracted to invest in its production. Over 90% of the farmers in Arumeru cultivated the black Lablab, with an overall average of 62.3% in black Lablab cultivation in the four study areas (Table 1).

In the Same district, Lablab is the most profitable cash crop (Same District Council, 2018). The study further suggested that the harvested Lablab beans per season significantly (p = 0.01) contributed to the farmers' livelihoods (Table 3). In addition, the collected Lablab beans per season significantly (p = 0.01) contributed to the farmers' livelihoods (Table 3). Notably, the revenue generated by Lablab per indicator was satisfactory (Table 2). A kilogram of Lablab beans, for example, cost TZS 4000–5000 (US$ 1.7–2.16) in the Arumeru district in northern Tanzania, whereas a kilogram of common beans costs TZS 1800–3000 (US$ 0.78–1.29). Similarly, in the Arusha region, Lablab beans could fetch TZS 120,000 (US$ 120) for 120 kg, compared to US$ 18 for an equivalent kg of maize (Shetto and Owenya, 2007). However, based on the five asset categories, financial assets accounted for approximately 30% of farmer revenues (Figure 6). Farmers' lives were also supported by 40% of their earnings from other crops (Table 3).

Physical assets included household goods purchased/improved using Lablab output earnings (Appendix 1). The revenue helped the farmers to repair or build homes and furnish them, buy cell phones, and use motorcycles (*boda-boda*) for transportation. Farmers who owned *boda-bodas* provided transportation services and increased their family incomes. The harvested Lablab was statistically significant (p ≤ 0.01) per season (Table 3), although farming inputs such as pesticides on average showed high consumption of Lablab income (58%) (Table 2). Lablab's output accounted for roughly 40% of total output in terms of tangible assets (Figure 6). However, as per the individual indicators in Table 2, farmers' lives were likely to be less supported. Gathering such multiple indicators on the wider perspective in agricultural aspects relative to rural livelihoods can expose detailed data that include income, food security, poverty, and rural relations for the future improvement of smallholder farmers' lives (Van Wijk et al., 2020).

Social assets linked Lablab farmers to a variety of social networks. Social networks are critical components of agricultural systems (Darnhofer and Vienna, 2016). The present results showed that social assets contributed about 30% to farmers' livelihoods (Figure 6). According to an indication of friends, relatives, and neighbors, social networking only had a 19% influence on support (Table 2). An elderly man from Arumeru explained that: "The Lablab business is less open, which leads to win and lose situations depending on the information acquired concerning market prices." This could account for the low Lablab profits among the farmers as a result of impaired communication which may have limited marketing opportunities. According to Halberg and Muller (2012), social networks are a powerful supporter of the development of social capital in rural communities. Furthermore, 39% of the farmers used cell phones as communication tools when looking for Lablab markets (Table 2). Although social assets are valuable instruments for farmers to engage with social groups, credit and savings organizations, or government officials to acquire wider information on livelihoods (FAO & ILO, 2009), the smallholder Lablab farmers in the present study were shown to be under-exposed.

### 3.3 Barriers faced by Lablab smallholder farmers

**Figure 7:**
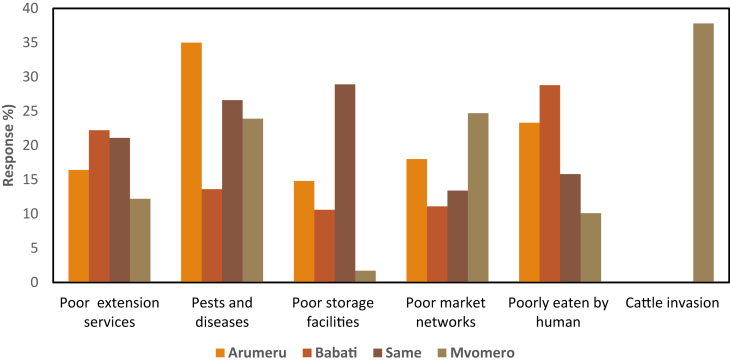
Barriers to Lablab production facing the smallholder farmers across the study sites.

Farming Lablab was critical in improving smallholder lives, but the farmers faced barriers that inhibited crop improvement. Figure 7 displays the major impediments to Lablab development in the study districts with variations depending on the study site.

Cattle invasion, for example, was most severe in the Mvomero district (37.8%), due to pastoralists' illegal cattle grazing on farmers' lands. Lablab beans, particularly the dominant black color accessions were underutilized as human food (Figure 3), hence less marketed in the country (Figure 7). Also, farmers' income was reduced due to a lack of unreliable markets for Lablab beans. This could be due to poor social network factors in the study sites (Table 2). Poorly organized and coordinated crop market linkages can result in high operating costs, volatile costs, unrealistic markets, and lower returns to farmers (Rusike et al., 2013). As a result, immediate and long-term solutions in Lablab production are needed to improve smallholder farmers’ livelihoods (Darnhofer, 2020).

## 4 Conclusions

This study assessed the LVC among Tanzanian smallholder farmers and its contribution to the farmers’ livelihoods. Due to their external marketability, black Lablab beans were the most cultivated but rarely consumed. Most crop seeds were recycled from previous harvests, relatives, or friends (67.7%). Harvesting was mostly manual by picking dry pods (61.6%), primarily in Arumeru (67.3%) and Babati (65.2%). However, the farmers despised this practice because it was time-consuming and costly. The farmers were the primary actors throughout the LVC, with traders only engaged in Lablab marketing. The chain contributed greatly to natural (70%) and human assets (50%), but less to physical (around 40%), social (30%), and financial assets (30%). Farmers' limited marketing exposure was associated with farm-gate selling at low prices immediately after harvesting, thus low-profit margins. Since Lablab is a climate-smart crop that can boost smallholder farmers' livelihoods, the study recommends interventions such as Lablab market-based reliability, openness, and accessibility; link of non-black accessions to the market; and support for smallholder farmers' access to quality seeds, extension services, and financial credit. The main limitation of the present study is that it did not assess value addition on Lablab beans for human consumption. Nevertheless, processing plants/warehouses can be established to add value to the crop, attract customers, and increase income. Otherwise, this can also facilitate the entry of Lablab value-added products into international markets, which will likely help smallholder farmers increase profit margins and improve farm gate prices for sustainable livelihoods. With the help of policymakers, researchers, and investors, the underutilized drought-tolerant Lablab can greatly contribute to farmers' livelihoods for a sustainable future.

## Declarations

### Author contribution statement

All authors listed have significantly contributed to the development and the writing of this article.

### Funding statement

Ms Josephine Minde was supported by Centre for Research, Agricultural Advancement, Teaching Excellence and Sustainability in Food and Nutrition Security (CREATES- FNS) partial scholarship funds for data collection [P.262/T17].

### Data availability statement

Data will be made available on request.

### Declaration of interests statement

The authors declare no conflict of interest.

### Additional information

No additional information is available for this paper.

## Figures and Tables

**Appendix 1:** Developed indicators (DI), with definitions and expected relationships

Livelihood assets	DI	Definition	Reference	Expected relationships in the farmers' household
Natural asset	Land and/or Livestock ownership	Land owned for agriculture	(Paudel Khatiwada et al., 2017a, Paudel Khatiwada et al., 2017b)	Agricultural activities; Collateral
		Value of livestock and their products	(Neudert et al., 2020); (Van Wijk et al., 2020)	A resource flow to various dimension of living
Human asset	Education	Schooling years	(Neudert et al., 2020)	Competence (knowledge & skills).
	Farming experience	Number of years involved in farming	(Kraaijvanger et al., 2016)	More skills and knowledge.
	Training/Advice	Participation in training/advice related to agriculture	(Huang et al., 2015); (de Satgé et al., 2002)	More exposure to farming activities
	Family size	Total household members	(Paudel Khatiwada et al., 2017a, Paudel Khatiwada et al., 2017b)	Bigger family sizes of working age are beneficial
Physical asset	Production tools	Availability of production tools	(de Satgé et al., 2002)	Expected to follow commercial agriculture/Simplify farming activity.
	Transport facilities	Transportation capability	(Seville et al., 2011)	Ease goods transportation.
	Household and its goods	Counted valued non-agricultural assets resulted from farming	(de Satgé et al., 2002)	Expected to add more worth to the household.
	Access to market	Distance in kilometer/s to the nearest market	(de Satgé et al., 2002)	Closer to the market/passable roads simplify marketing.
	Agricultural inputs	Access and affordable	(Van Wijk et al., 2020)	Improve production hence commercialization
Social Asset	Social support Friend/relatives	Ways of supporting each other	(Østerberg et al., 2017)	Relationships of trust draw people to profitable farm and non-farm activities.
	Communication (networks)	Communication facilities	(Paudel Khatiwada et al., 2017a, Paudel Khatiwada et al., 2017b)	Social resources networks updated shared information.
Financial Asset	Income from Lablab production	Money collections from Lablab business.	(Seville et al., 2011)	Financial resources from Lablab farming contribute to different livelihood options.
	Income not from Lablab	Collection from other sources	(Paudel Khatiwada et al., 2017a, Paudel Khatiwada et al., 2017b)	More addition to livelihood activities.
